# Anti-Inflammatory Effect of *Channa micropeltes* Extract in Angiogenesis of Diabetes Mellitus Wound Healing

**DOI:** 10.1900/RDS.2022.18.166

**Published:** 2022-12-31

**Authors:** Maharani Laillyza Apriasari, Dewi Puspitasari, Juliyatin Putri Utami

**Affiliations:** 1 Department of Oral Medicine, Faculty of Dentistry, Universitas Lambung Mangkurat, Banjarmasin, Kalimantan Selatan, Indonesia,; 2 Department of Dental Material, Faculty of Dentistry, Universitas Lambung Mangkurat, Banjarmasin, Kalimantan Selatan, Indonesia,; 3 Department of Biomedicine, Faculty of Dentistry, Universitas Lambung Mangkurat, Banjarmasin, Kalimantan Selatan, Indonesia.

**Keywords:** *channa micropeltes*, NF-κB, VEGF, neovascular ·wound healing, diabetes mellitus, retinopathy

## Abstract

**OBJECTIVE:**

*Channa micropeltes* extract contains albumin and omega-6 which possess antioxidant and anti-inflammatory agents that can promote macrophages in the wound healing process associated with diabetes mellitus (DM). In this study, we analyzed Nuclear Factor kappa B (NF-κB) and Vascular Endothelial Growth Factor (VEGF) expression as well as neovascular cells in the inflammatory stage of DM wound healing.

**METHODS:**

The 24 males *Rattus novergicus* were divided into 3 groups that were 20% *Channa micropeltes* ointment (Group I), 10% *Channa striata* extract ointment (Group II), and placebo ointment as a control (Group III). Ointments were applied 3 times daily.

**RESULTS:**

The highest expression of NF-κB was obser ved in Group III on Day 4 (15.50 ± 2.38), and the lowest was in treatment of Group I and Group II on Day 8 (4.75 ± 0.96). The highest expression of VEGF was obser ved in Group I on Day 8 (14.75 ± 0.96), and the lowest was Group III on Day 4 (7.00 ± 1.41). The highest count of neovascular cells was obser ved in Group I on Day 8 (11.00 ± 2.16), and the lowest was in Group III on Day 4 (5.50 ± 0.58).

**CONCLUSIONS:**

*Channa micropeltes* has an anti-inflammator y effect by regulating NF-κB expression and elevating VEGF expression in the angiogenesis process of DM wound healing.

## 1 Introduction

Indonesia ranks sixth among countries with a high prevalence of diabetes mellitus (DM). In an uncontrolled state, DM will result in various oral problems such as xerostomia, candidiasis, stomatitis, gingivitis and periodontitis. The healing of such conditions is often complicated due to hyperglycemia that initiates chronic inflammation [1-3]. Previous study revealed that *Channa micropeltes* ointment at 20% concentration or *Channa striata* ointment at 10% concentration applied topically can accelerate wound healing in a DM rat model. Both species are categorized in the same genus and contain albumin as well as omega-6 that acts as an antioxidant and anti-inflammatory [3,4]. As an antioxidant, *Channa micropeltes* extract elevates superoxide dismutase (SOD) activity and lowers malondialdehid (MDA) level on Day 7 [2]. As an anti-inflammatory, the topical application of *Channa micropeltes* at 20% concentration on the back skin of a diabetic rat model can increase the number of macrophage and lymphocyte cells on Day 8 and gradually reduce it on Day 14 [1].

Macrophage is the key inflammatory process in wound healing. Reduction in macrophage number at the end of inflammatory stage demonstrates tissue recovery by producing growth factors and cytokines and inducing as well as terminating angiogenesis [5,6]. Macrophage is also produced Nuclear Factor kappa B (NF-κB) that regulates the inflammatory response of metabolic disease such as DM. A state of hyperglycemia in DM will increase reactive oxygen species (ROS) and advanced glycation end products (AGEs) that elevate chronic inflammation through the activation of NF-κB. This will change vascular endothelial growth factor (VEGF) expression that will generate the damage to blood vessels in the angiogenesis process [7,8].

The extract of *Channa micropeltes* has shown to promote wound healing on the skin of diabetic rat model by reducing macrophage number at the end of inflammatory stage [3]. There has been no study that explores the effect of *Channa micropeltes* application on NF-κB, VEGF, and neovascular cells that are pivotal components in the DM wound healing process. Based on that, a study to analyze the expression of NF-κB, VEGF and neovascular cells number at the inflammatory stage of the DM wound healing process was warranted.

## 2 Methods

This study was an experimental laboratory research study incorporating a post-test only control group design. It was approved by Ethical Clearance Committee, Faculty of Dentistry, Universitas Lambung Mangkurat, Banjarmasin, South Kalimantan, Indonesia, with number 111/KEPKG-FKGULM/EC/ III/2020.

### 2.1 Manufacturing Channa micropeltes and Channa striata extract

Preparing both *Channa micropeltes* and *Channa striata* extract used fresh fish weighing 600-1000 grams. Each extract was later steamed in a pan for 25-35 minutes at a temperature of 60^o^ Celsius. The flesh was enclosed with flannelette and pressed in a hydraulic device. Furthermore, *Channa micropeltes* and *Channa striata* were centrifuged for 15 minutes at a speed of 6000 rpm. Each extract was kept inside a dark glass bottle and then covered with aluminum foil and clean pack.

### 2.2 Formulation of Channa micropeltes and Channa striata ointment

Adeps lanae (Asian chemicals, Semarang) in a weight of 16.875 grams and vaselin flavum (PT. Brataco, 1295578) in a weight of 23.125 grams were used in the formulation of the *Channa micropeltes* ointment. Meanwhile, a combination of 16.875 grams adeps lanae and 28.125 grams vaselin flavum were combined in the formulation of *Channa striata* ointment. Adeps lanae was initially poured into different tubes for each extract and later added gradually with either *Channa micropeltes* at 20% concentration or *Channa striata* at 10% concentration. After the extract was fully absorbed by adeps lanae, the mixture was then mashed to obtain a homogenous consistency. Subsequently, the composition was further mixed with vaselin flavum and mashed again until homogenous.

### 2.3 In vivo study

This study included 2-3 months old male Wistar (*Rattus novergicus*) rats (weight, 250-300 grams) obtained from an animal laboratory at the Faculty of Medicine, University of Lambung Mangkurat. The 24 rat specimens were kept in cages, and the temperature and humidity were set within ±25 °C and 60%, respectively. They were fed standard BRII, and they had access to boiled water *ad libitum*. Rats with hyperglycemia were obtained by injecting streptozotocin (STZ) at 35 mg/kg dosage until the blood glucose level was over 126 mg/dL-1; non-diabetic rats were ones without intervention. All animals were divided into 3 treatment groups consisting of 20% *Channa micropeltes* extract ointment, 10% *Channa striata* extract ointment, and placebo ointment as control. Each substance was applied topically 3 times daily (every 6-8 hours). An incisional wound was made on the back of the rats with 1 cm length and 1 mm depth using sterile scalpel under inhaled anesthesia of 5 ml diethyl ether.

After the 4th and 8th day of application, rats were euthanized by inhaling a lethal dosage of diethyl ether. The back skin was then biopsied for histopathology examination using hematoxylin eosin (HE) to evaluate macrophages and neovascular cells, and immunohistochemistry (IHC) to evaluate NF-κB and VEGF. The number of macrophages and neovascular cells were calculated in 3 different field locations using a light microscope (Olympus, WA) at 400 magnifications and subsequently calculated for its average. IHC staining was performed using anti-mouse NF-kB monoclonal antibody (Santa Cruz Biotechnology Inc, Santa Cruz, CA, NF-kB p65 (F-6): sc 8008) and anti-mouse VEGF monoclonal antibody (Santa Cruz Biotechnology Inc, Santa Cruz, CA, VEGF (C1): sc 7269). Positivity NF-κB expression was defined as only distinct nuclear immunostaining, which is considered as activated NF-κB in the studied field at 100 magnifications.

### 2.4 Data analysis

The results were analyzed using a 2-way analysis of variance parametric test based on the Shapiro-Wilk normality test and Levene’s variance homogeneity test. The results showed normal data distribution and homogenous data variances. Consequently, further analysis by means of a *post hoc* Bonferroni test was conducted with a statistical significance of p < 0.05.

## 3 Results

Table 1 shows the results of NF-κB analysis. The highest expression of NF-κB was observed in the control group (Group III) on Day 4 (15.5 ± 2.38), and the lowest was in the treatment of 20% *Channa micropeltes* extract ointment (Group I) (4.75 ± 0.96) and 10% *Channa striata* extract ointment (Group II) (5.75 ± 1.71) on Day 8. The expression of NF-κB between Group I and 10% *Channa striata* extract ointment (Group II) treatment groups did not show any difference (p > .05; Table 1). The statistical significance value between treatment group and day was p > 0.05, demonstrating no interaction between treatment groups and days on NF-κB expression.

**Table 1.** The expression of NFkB, expression of VEGF and count of neovascular cells

Group			Mean ± SD (cells)			
	Expression of NFkB		Expression of VEGF		Count of Neovascular	
	Day 4 Day 8		Day 4 Day 8		Day 4 Day 8	
Channa micropeltes (Group 1)	7.00 ± 1.41^A^	4.75 ± 0.96^A^	11.75 ± 1.71^C^	14.75 ± 0.96^C^	7.75 ± 0.96^C^	11.00 ± 2.16^C^
Channa striata (Group II)	7.7 5± 1.26^A^	5.75 ± 1.71^A^	10.50 ± 1.29^B^	12.50 ± 1.29^B^	6.25 ± 0.96^B^	9.00 ± 0.816^B^
Control (Group III)	15.50 ± 2.38^B^	10.00 ± 2.58^B^	7.00 ± 1.41^A^	9.75 ± 0.96^A^	5.50 ± 0.58^A^	6.25 ± 0.96^A^

Note.

Abbreviations: The different superscript character in each variable shows the differences for each group (p < 0.05).

A in Expression of NFkB has value p = 0.044

B in Expression of NFkB has value p = 0.000

A, B and C in Expression of VEGF has value p = 0.000

A, B and C in Count of Neovascular has value p = 0.000

The highest expression of VEGF was observed in Group I (14.75 ± 0.96) on Day 8, whereas the lowest was in Group III (7.00 ± 1.41) on Day 4. The expression of VEGF showed statistically significant differences in all groups (p < 0.05; Table 1). The significance value between treatment groups and days was p > 0.05, demonstrating no interaction between treatment group and day on VEGF expression.

The highest count of neovascular cells was observed in Group I (11.00 ± 2.16) on Day 8, while the lowest was in Group III (5.50 ± 0.58) on Day 4. The count of neovascular cells showed statistically significant differences in all groups (p < 0.05; Table 1). The statistical significance value between treatment groups and days was p > 0.05, demonstrating no interaction between treatment groups and days on the number of neovascular cells.

## 4 Discussion

DM is characterized with an increase in blood glucose level that induces glycation reaction. This process will result in amadory production to formulate toxic proteins (AGEs). Interaction between AGEs and a receptor advanced glycation end product (RAGE) will increase the signal for nicotinamide adenine dinucleotide phosphate (NADPH) oxidase which produces superoxide anion. This process elevates the production of reactive oxygen species (ROS) which are the key for molecular signaling as well as the development of inflammatory disorders such as DM. Excessive production of ROS will complicate the healing process of wounds in DM [2,9].

*Channa micropeltes* contains albumin and omega-6 fatty acid. Albumin can decrease ROS by cutting chained oxidative reaction in the ROS formation’s process.

Albumin can bind metal ions and also catch oxygen that processing hydrogen peroxide into non radical compound. Omega-6 fatty acid, especially arachnodic acid are the keys to anti-inflammatory processes. It plays the role in stimulating macrophages to release growth factors, such as VEGF. Arachidonic acid will be metabolized through an enzymatic mechanism such as the 5-lipoxygenase and cyclo-oxygenase pathways that produce leukotrienes, prostaglandins, and thromboxane A2. These can stimulate cell migration and new local vascularization in the wound healing process of DM [4,10].

**Figure 1.**
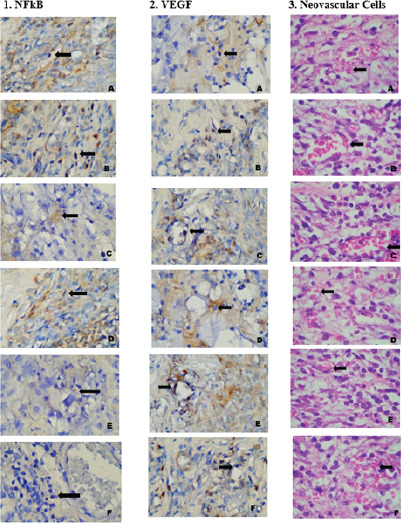
Macrophage expression of NF-κB on the control group, Neovascular’s expression of VEGF on the control group, and Neovascular cell’s count on the control group. Note: Macrophage’s expression of NF-κB on control group (A), Channa micropeltes extract concentrations of 20% (B), Channa striata extract concentrations of 10% (C) on Day 4. Macrophage’s expression of NF-κB on control group (D), Channa micropeltes extract concentrations of 20% (E), Channa striata extract concentrations of 10% (F) on Day 8. 2. Neovascular’s expression of VEGF on control group (A), Channa micropeltes extract concentrations of 20% (B), Channa striata extract concentrations of 10% (C) on Day 4. Neovascular’s expression of VEGF on control group (D), Channa micropeltes extract concentrations of 20% (E), Channa striata extract concentrations of 10% (F) on Day 8. 3. Neovascular cell’s count on control group (A), Channa micropeltes extract concentrations of 20% (B), Channa striata extract concentrations of 10% (C) on Day 4. Neovascular cell’s count on control group (D), Channa micropeltes extract concentrations of 20% (E), Channa striata extract concentrations of 10% (F) on Day 8.

In previous studies, *Channa micropeltes* extract ointment at 20% concentration and *Channa striata* extract ointment at 10% concentration were shown to promote the wound healing process in DM. *Channa micropeltes* and *Channa striata* are categorized in the same genus. Both species contain albumin, the secondary antioxidant that can bind metal ion in ROS formation [1,2]. ROS induces an inflammatory response through the activation of Nuclear Factor kappa B (NF-κB). NF-κB signal is the main key of chronic inflammation in DM [7,8]. This is demonstrated by the study result on Day 4 that reveals the highest expression of NF-κB in control group, while the result for 20% *Channa micropeltes* extract application was comparable to 10% *Channa striata* extract application (Figure 1).

Our study result on Day 8 demonstrates the reduction of NF-κB expression in both *Channa micropeltes* extract ointment at 20% concentration and *Channa striata* extract ointment at 10% concentration when compared to the control. Topical application of *Channa micropeltes* extract at 20% concentration or *Channa striata* extract at 10% concentration may reduce excessive ROS, thereby suppressing NF-κB expression. Both extracts possess potential as natural substances that may inhibit the expression of NF-κB. Previous studies reveal that the impediment of pro-inflammatory NF-κB from therapeutical application of several natural and synthetic ingredients will be a good target to manage vascular complication in DM [7]. A prolonged inflammatory response can be resolved by the inhibition of NF-κB. As an anti-inflammatory substance, *Channa micropeltes* will reduce inducible nitric oxide synthase (iNOS) and cyclooxygenase 2 (COX2) that suppress the NF-κB gene regulator. This will prevent prolonged inflammation in the wound healing process of DM [11,12].

Excessive activation of NF-κB will cause abnormal DNA transcription which includes various gene expression of vascular complications occurring in VEGF, Platelet Derived Growth Factor (PDGF), Endothelin-1 (ET-1) and Transforming Growth Factor beta (TGF-β) that cause vascular cell damage [7]. VEGF as pro-angiogenic modulators encounter down regulation in DM, disturbing the angiogenesis process [13,14]. Prior studies demonstrate Vascular Endothelial Growth Factor A (VEGF-A) protein and messenger ribonucleic acid (mRNA) level in the wound of a diabetic rat model which shows reduction when compared to the group with a normal wound. DM leads to the decrease of angiogenesis in wound healing, so that it lessens vascular and capillary density [13].

The angiogenic effect is initiated by VEGF-A binding to Vascular Endothelial Growth Factor Receptor-2 (VEGFR-2). Angiogenesis stimulation through PI3KAkt- eNOS will cause endothelial cell to migrate, proliferate, and differentiate. Molecular signals will be commenced by phosphatidylinositol 3 kinase from serine/threonine kinase Akt/protein kinase B. Akt/ PKB through the phosphorylation of endothelial nitric oxide synthesis on Ser 1177, and will stimulate NO production, vasodilatation, and endothelial cell migration [15,16]. This result presents that *Channa micropeltes* extract ointment at 20% concentration increases the highest expression of VEGF when compared to *Channa striata* extract ointment at 10% concentration or the control on Day 8. This exhibits the potential of *Channa micropeltes* extract ointment at 20% concentration to promote angiogenesis on the DM wound healing process.

Previous study by Carabelly et al. [1] reveals that the application of *Channa micropeltes* extract ointment at 20% concentration may elevate macrophage number on Day 8 and reduce them on Day 14. Macrophage regulates angiogenesis signals in neovascular along the formation of granulation tissue process [17]. This concept is in accordance with this study as the number of macrophages was observed the highest on Day 8 and followed by the increase of neovascular cell number by the application of *Channa micropeltes* extract ointment at 20% concentration on Day 8. The highest number of neovascular cells also was observed in the application of *Channa micropeltes* extract ointment at 20% concentration when compared to *Channa striata* extract ointment at 10% concentration or the control in Day 8. This study did not continue to further days to limit the parameters.

We can conclude that the application of *Channa micropeltes* extract ointment at 20% concentration on the wound of a diabetic rat model can reduce the expression of NF-κB. The anti-inflammatory effect of *Channa micropeltes* can elevate the expression of VEGF and the number of neovascular cells in angiogenesis process of diabetic wound healing. Our findings require further research using different parameters, adding time of evaluation, and using larger experimental animals.

## Abbreviations

AGEs: Advanced Glycation End products

COX2: Cyclooxygenase 2

DM: Diabetes mellitus

ET-1: Endothelin-1

HE: Hematoxylin eosin

IHC: Immunohistochemistry

iNOS: Nitric oxide synthase

MDA: Malondialdehid

mRNA: Messenger ribonucleic acid

NADPH: Nicotinamide adenine dinucleotide phosphate

NF-κB: Nuclear Factor Kappa Beta

PDGF: Platelet Derived Growth Factor

RAGE: Receptor advanced glycation end product

ROS: Reactive oxygen species

SOD: Superoxide dismutase

STZ: Streptozotocin

VEGF: Vascular Endothelial Growth Factor
